# Circulating extracellular particles from severe COVID-19 patients show altered profiling and innate lymphoid cell-modulating ability

**DOI:** 10.3389/fimmu.2023.1085610

**Published:** 2023-05-03

**Authors:** Dorian Forte, Roberto Maria Pellegrino, Sara Trabanelli, Tommaso Tonetti, Francesca Ricci, Mara Cenerenti, Giorgia Comai, Pierluigi Tazzari, Tiziana Lazzarotto, Sandra Buratta, Lorena Urbanelli, Ghazal Narimanfar, Husam B. R. Alabed, Cristina Mecucci, Gaetano La Manna, Carla Emiliani, Camilla Jandus, Vito Marco Ranieri, Michele Cavo, Lucia Catani, Francesca Palandri

**Affiliations:** ^1^ Department of Medical and Surgical Sciences (DIMEC), Institute of Hematology ‘Seràgnoli’, University of Bologna, Bologna, Italy; ^2^ Department of Chemistry, Biology and Biotechnology, Biochemistry and Molecular Biology Section, University of Perugia, Perugia, Italy; ^3^ Department of Pathology and Immunology, Faculty of Medicine, University of Geneva, Geneva, Switzerland; ^4^ Ludwig Institute for Cancer Research, Lausanne Branch, Lausanne, Switzerland; ^5^ Department of Medical and Surgical Sciences (DIMEC), University of Bologna, Bologna, Italy; ^6^ Anesthesia and Intensive Care Medicine, IRCCS Azienda Ospealiero-Universitaria di Bologna, Bologna, Italy; ^7^ Immunohematology and blood bank, IRCCS Azienda Ospedaliero-Universitaria di Bologna, Bologna, Italy; ^8^ Nephrology, Dialysis and Renal Transplant Unit, IRCCS Azienda Ospedaliero-Universitaria di Bologna, Bologna, Italy; ^9^ Microbiology Unit, IRCCS Azienda Ospedaliero-Universitaria di Bologna, Bologna, Italy; ^10^ Department of Medicine and Surgery, Center for Hemato-Oncology Research (C.R.E.O.), University of Perugia, Perugia, Italy; ^11^ Istituto di Ematologia “Seràgnoli”, IRCCS Azienda Ospedaliero-Universitaria di Bologna, Bologna, Italy

**Keywords:** COVID-19, SARS-CoV-2, extracellular vesicles and particles, innate lymphoid cells, type 2 innate lymphoid cell, lipidomic

## Abstract

**Introduction:**

Extracellular vesicles (EVs) and particles (EPs) represent reliable biomarkers for disease detection. Their role in the inflammatory microenvironment of severe COVID-19 patients is not well determined. Here, we characterized the immunophenotype, the lipidomic cargo and the functional activity of circulating EPs from severe COVID-19 patients (Co-19-EPs) and healthy controls (HC-EPs) correlating the data with the clinical parameters including the partial pressure of oxygen to fraction of inspired oxygen ratio (PaO2/FiO2) and the sequential organ failure assessment (SOFA) score.

**Methods:**

Peripheral blood (PB) was collected from COVID-19 patients (n=10) and HC (n=10). EPs were purified from platelet-poor plasma by size exclusion chromatography (SEC) and ultrafiltration. Plasma cytokines and EPs were characterized by multiplex bead-based assay. Quantitative lipidomic profiling of EPs was performed by liquid chromatography/mass spectrometry combined with quadrupole time-of-flight (LC/MS Q-TOF). Innate lymphoid cells (ILC) were characterized by flow cytometry after co-cultures with HC-EPs or Co-19-EPs.

**Results:**

We observed that EPs from severe COVID-19 patients: 1) display an altered surface signature as assessed by multiplex protein analysis; 2) are characterized by distinct lipidomic profiling; 3) show correlations between lipidomic profiling and disease aggressiveness scores; 4) fail to dampen type 2 innate lymphoid cells (ILC2) cytokine secretion. As a consequence, ILC2 from severe COVID-19 patients show a more activated phenotype due to the presence of Co-19-EPs.

**Discussion:**

In summary, these data highlight that abnormal circulating EPs promote ILC2-driven inflammatory signals in severe COVID-19 patients and support further exploration to unravel the role of EPs (and EVs) in COVID-19 pathogenesis.

## Graphical Abstract

Created with BioRender.com.

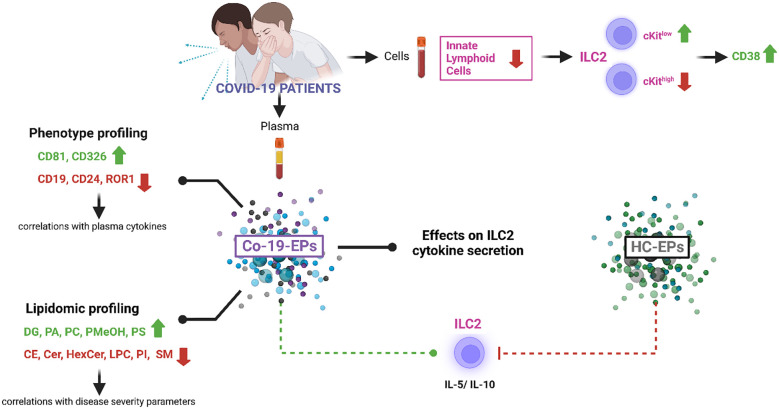

## Introduction

Coronavirus disease 2019 (COVID-19) is caused by Severe Acute Respiratory Syndrome Coronavirus 2 (SARS-CoV-2). Although most infected patients have mild to moderate symptoms or are even asymptomatic, older patients and those with pre-existing chronic diseases (e.g., hypertension, diabetes, obesity) are at greater risk of developing serious complications, such as pneumonia, cytokine storm and multiple organ failure (1, 2).

The critical production of (pro)inflammatory cytokines and chemokines detected during COVID-19 infection is mainly responsible for the broad and uncontrolled tissue damage observed in patients (3–11). Along with cytokine storm, immune dysregulation with quantitative abnormalities and impaired functional activity of innate and adaptive immune cells including innate lymphoid cells (ILC), monocytes/macrophages, dendritic cells, NK cells and T/B cells has been observed in COVID-19 patients (6, 12–14).

Specifically, ILC play a pivotal role in immune surveillance and form the front line of immune defense. Natural killer cells (NK), one of the ILC subsets belonging to the group 1 ILC (15), are known to perform lytic functions, instead ILC1, ILC2, and ILC3 subsets have mainly helper functions through secretion of Type 1, Type 2 and Type 17 cytokines, respectively (16, 17). In peripheral blood, an additional subset of ILCs has been identified and named ILC precursor (ILCP) because of its ability to give rise, both *in vitro* and *in vivo*, to all ILC subsets (18). ILC are largely depleted from the circulation of COVID-19 patients (13, 19). The remaining circulating ILC reveal decreased frequencies of ILC2 in severe COVID-19, with a concomitant decrease of ILCP, as compared with HC. ILC2 and ILCP show an activated phenotype with increased CD69 expression which is positively correlated with the levels of IL-6 and IL-10, while frequencies of ILC subsets are correlated with clinical and biochemical laboratory parameters associated with disease severity (19, 20). However, the mechanism(s) leading to altered ILC activation and/or function in COVID-19 is yet to be determined.

Extracellular vesicles (EVs) are lipid bilayer structures with a key role within the inflammatory network. They are released from a broad variety of cells during homeostasis and cell activation with pleiotropic effects on cell-cell signaling, by transferring bioactive molecules into recipient cells or by regulating the downstream signal cascades of receptors on target cells. Based on size and biogenesis, small and large EVs can be identified. EVs contain functionally relevant biomolecules such as proteins, nucleic acids and lipids. They have been detected in various biological fluids including blood (21–23).

Recently, it has been described that EVs are involved in SARS-CoV-2 infection (24, 25). Circulating platelet-derived EVs have been described to be increased in COVID-19 patients (26–28). EVs may be also involved in 1) virus entry and spreading through the expression of the SARS-CoV-2 receptors angiotensin-converting enzyme (ACE)-2 (29) and CD9 (30, 31), 2) immune dysregulation, cytokine storm development and maintenance (30), 3) inflammation and thrombosis (32). In addition, a diagnostic (33) and prognostic (34, 35) role of EVs has been suggested.

In this work, considering the EV identity defined by MISEV 2018 (22) and their heterogeneity, we collectively referred to them as extracellular particles (EPs). To further understand the impact of EPs on COVID-19 infection, here we studied the lipid cargo/phenotype of EPs in COVID-19 patients and the functional activity of circulating EPs on ILC in severe COVID-19 patients as reported by the Graphical Abstract.

## Materials and methods

### Patients’ characteristics

Ten COVID-19 patients, admitted to the Intensive Care Unit of the IRCCS Azienda Ospedaliero-Universitaria di Bologna, were enrolled in the study. Patients were diagnosed with COVID-19 using reverse-transcriptase polymerase chain reaction viral detection of oropharyngeal or nasopharyngeal swabs. We considered only critical patients for this study with respiratory failure and admitted to the intensive care unit with the need for mechanical ventilation.

Demographic and laboratory findings of all recruited COVID-19 patients are summarized in **Table 1**. In addition to age, sex, hospitalization duration, clinical outcome and date/timing of peripheral blood sample collection, patients were assessed for the presence or the absence of the following pre-existing medical condition: lung disease (asthma, chronic obstructive pulmonary disease (COPD)), heart disease (coronary artery disease, heart failure), peripheral vascular disease, hypertension, diabetes, obesity (BMI >30), kidney disease, autoimmune disorders, cancer, chemotherapy for cancer. Laboratory parameters at the time of sample collection were analyzed and the serum levels of ferritin, C-reactive protein, D-dimer, and lactate dehydrogenase were recorded for each patient as well as the number of white blood cells (WBC), platelets (PLT), hematocrit and hemoglobin.

**Table 1:** Clinical and demographic characteristics of COVID-19 patients and HC.

		COVID-19 PATIENTS	HEALTHY CONTROLS
**Number**		10	10
**Age**	Years, median(range)	67 (56–76)	67.5 (43–72)
**Female/Male**	n°	2/8	3/7
**Comorbidities**	Arterial hypertension, n° (%)	5 (50%)	NA
	Peripheral artery disease, n° (%)	1 (10%)	NA
	COPD, n° (%)	2 (20%)	NA
	Diabetes, n° (%)	2 (20%)	NA
	Acute myocardial infarction, n° (%)	1 (10%)	NA
	Others, n° (%)	4 (40%)	NA
**Treatment at time of sampling**	Anticoagulant, n° (%)	10 (100%)	
	Antibiotics, n° (%)	9 (90%)	
	Glucocorticoids, n° (%)	5 (50%)	
**At time of sampling**	Platelets x 10^9^/L, median(range)	239 (139–512)	200.5 (154–261)
	White Blood Cells x 10^9^/L, median(range)	7.9 (3.8-15.6)	5,9 (4.0-9.0)
	c-Reactive Protein (CRP), median(range)	2.6 (0.33-13.9)	NA
	PaO_2_, mm Hg median (range)	83 (57–132)	NA
	PaCO_2_, mm Hg median (range)	49.5 (40-76)	NA
**Ventilation mode at time of blood sampling**	Pressure/volume control, n° (%)	3 (30%)	NA
	Pressure support, n° (%)	5 (50%)	NA
	Spontaneous, n° (%)	2 (20%)	NA
**Intensive Care Unit stay**	Days at time of sampling, median(range)	28.5 (8-43)	NA
**PaO_2_/FiO_2_ score**	median(range)	186 (113-330)	NA
**SOFA score**	median(range)	2.5 (1-8)	NA

Regarding COVID-19 disease severity parameters, the partial pressure of oxygen to fraction of inspired oxygen ratio (PaO_2_/FiO_2_) and the sequential organ failure assessment (SOFA) score were recorded. The use of specific COVID-19-targeted treatment has been also reported. Specimens from anonymous pre-screened healthy blood controls (HC; n=10), matched for sex and age, were collected from the blood donor center. This study was approved by the Ethics Committee of the IRCSS Azienda Ospedaliero-Universitaria di Bologna and written informed consent was obtained from all patients/controls enrolled in the study.

### Blood sample collection and plasma preparation

Venous EDTA-blood was kept vertically at room temperature and processed within 1 hour. After the first centrifugation of 15 min 2,500 x g at room temperature, plasma was collected and subjected to second centrifugation of 15 min 2,500 x g at room temperature to obtain platelet-free plasma. Platelet-free plasma was then stored at -80°C until use.

### EP isolation

Samples were defrosted at room temperature and EP isolation was achieved by size-exclusion chromatography (SEC; qEVoriginal/70 nm Gen 2 Column, Izon) following the manufacturer’s instructions. In brief, the column was equilibrated with PBS before loading the sample (500 µl) on top of the column. Next, four fractions were collected after void volume. Then, where indicated, EP-enriched fractions were pooled for maximizing yield for downstream experiments using MWCO 30 kDa Amicon Ultra-2 Centrifugal Filters (Millipore, Merck, USA). Finally, all samples were used or stored at -80°C until use. The protein content of the EPs was determined using the Bradford assay according to the manufacturer’s instructions.

### EP lipid extraction and LC/MS Q-TOF analysis

The EP lipidome was quantified using an untargeted lipidomic approach. Specifically, lipids were extracted from EP samples according to the one-phase extraction method described by (36) with minor modifications. In brief, 18mL of MMC extraction solvent was prepared by adding 5mL of methanol (MeOH), 6mL of chloroform (CHCl_3_), 6mL of metil-t-butil etere (MTBE) and 1mL of Internal Standard mixture Splash I Lipidomix (Avanti Polar Lipids, USA) diluted 1:10 in MeOH. Each sample was added with 600 μl of MMC, vortexed for 10 seconds and shaken at 1600 rpm at 20°C in a T-Shaker (Euroclone). At the end, the tubes were centrifuged for 20 min at 16,000 x g at 4°C. The supernatant was transferred to a 1.5mL glass vial and flushed to dryness with a gentle stream of nitrogen. The residue was resuspended with 200 μl of a 9:1 mixture (MeOH/Toluene) and subjected to LC/MS Q-TOF analysis.

LC/MS Q-TOF analysis was carried out according to (37), after adaptation to the different instrumental configurations and using a 1260 Infinity II LC System coupled with an Agilent 6530 Q-TOF spectrometer (Agilent Technologies, Santa Clara, CA USA). Separation was carried out on a reverse phase C18 column (Agilent InfinityLab Poroshell 120 EC-C18, 3.0 × 100 mm, 2.7 µm) at 50°C and 0.6 mL/min flow. Mobile phase consisted of a mixture of water (A), Acetonitrile (can) (B), MeOH (C) and Iso-propanol (IPA) (D) all containing a concentration of 10 mM ammonium acetate and 0.2 mM of ammonium fluoride except for ACN. Gradient was time 0-1 min isocratic at A 27%, B 14%, C 24%, D 35%; time from 1 to 3.5min: linear gradient to A 12.6%, B 17.2%, C 27.2%, D 43%; time 3.5-10 min isocratic; time from 10 to 11 min: linear gradient to A 0%, B 20%, C 30%, D 50%; time 11-17 min isocratic; time 17-17.1 min: linear gradient to A 27%, B 14%, C 24%, D 35%; time 20 min: stop run. Spectrometric data were acquired in the 40-1700 m/z range both in negative and positive polarity. An iterative MS/MS acquisition mode on three technical replicates for each sample was used. The Agilent JetStream source operated as follows: Gas Temp (N2) 200°C, Drying Gas 10 L/min, Nebulizer 50 psi, Sheath Gas temp: 300°C at 12 L/min. MS/MS spectra were obtained using N2 at 30V CE.

Acquired raw data were processed using the MS-DIAL software (4.48) (38) to perform peak-picking, alignment, annotation and quantification. Lipid annotation and quantification were carried out according to the recommendations of Lipid Standard Initiative (39).

At the end of the workflow, a data matrix containing the concentration in nmol/mL of the annotated lipids distributed over various lipid classes was obtained. The tool LipidOne was used to perform an in-depth analysis in lipid compositions, called lipid building blocks (40). The volcano plot and network graphs were created with Excel (Microsoft) and Graph Editor (https://csacademy.com/app/graph_editor/) by processing the data obtained with LipidOne. MetaboAnalist 5.0 web platform was used to perform multivariate statistical and chemoinformatic analysis (41).

### MACSPlex

MACSPlex analysis was performed using the MACSPlex Exosome Kit, human (Miltenyi Biotec, Bergisch-Gladbach, Germany) according to the manufacturer’s instructions. Briefly, EP-enriched pool were diluted with MACSPlex buffer and MACSPlex Exosome Capture Beads were added. After overnight incubation at room temperature in agitation, MACSPlex Exosome Detection Reagent for CD9, CD63, and CD81 were added to each sample followed by incubation for 1 hour at room temperature. Flow cytometric analysis was carried out on a CytoFLEX flow cytometer followed by Kaluza Analysis 2.1 (Beckman and Coulter Life Sciences, CA, USA). Exosomal surface epitope expression (median APC fluorescence intensity) was then recorded. Median fluorescence intensity (MFI) was evaluated for each capture bead subsets and corrected by subtracting the respective MFI of blank control (PBS, vehicle) and normalized by the mean MFI of CD9, CD63, and CD81.

### Plasma cytokine concentration

Plasma from HC and COVID-19 patients were analyzed using BioLegend’s LEGENDplex™ bead-based immunoassays to quantify IL-2, IL-4, IL-5, IL-6, IL-9, IL-10, IL-13, IL-17A, IL-17F, IL-22, IFN-γ and TNF-α (Human Th Cytokine Panel (12-plex)) and to quantify IL-1β, IL-6, TNF-α, IP-10, IFN-λ1, IL-8, IL-12p70, IFN-α2, IFN-λ2/3, GM-CSF, IFN-β, IL-10 and IFN-γ (Human Anti-Virus Response Panel (13-plex)). A customized Human Th Cytokine Panel, including only IL-4, IL-5, IL-9, IL-10 and IL-13, was also used to quantify these cytokines in the supernatants of *in vitro* stimulated ILC2. The analyses were performed according to the manufacturer’s instructions.

### ILC phenotype, isolation and expansion

ILC were identified by flow cytometry using the following antibodies:

FITC-conjugated anti-CD3 (clone: UCHT1), anti-CD4 (clone: RPA-T4), anti-CD8 (lot: 276276, Immunotools), anti-CD14 (clone: HCD14), anti-CD15 (clone: HI98), anti-CD16 (lot: 7464017, Beckman Coulter), anti-CD19 (clone: HIB19), anti-CD20 (clone: 2H7), anti-CD33 (clone: HIM3-4), anti-CD34 (clone: 561), anti-CD203c (clone: NP4D6), anti-FcϵRI (clone: AER-37) all from Biolegend); BUV737-conjugated anti-CD56 (clone: NTAM16.2, BD); BV421-conjugated anti-CD127 (clone: A019D5, Biolegend); BV605-conjugated anti-CD117 (cKit, clone: 104D2, Biolegend); BUV395-conjugated anti-CRTH2 (clone: BM16, BD Biosciences). Total ILC were identified as Lineage^-^ (CD3^-^, CD4^-^, CD8^-^, CD14^-^, CD15^-^, CD16^-^, CD19^-^, CD20^-^, CD33^-^, CD34^-^, CD203c^-^, FcϵRI^-^), CD56^-^, CD127^+^ lymphocytes. From total ILC, ILC1 were identified as CRTH2^-^cKit^-^, ILC2s as CRTH2^+^cKit^+/-^ and ILCP as CRTH2^-^cKit^+^. The gating strategy is shown in **Supplementary Figure S1**.

ILC2 phenotype was analysed by using: APC-conjugated anti-CD69 (clone: FN50, BD Biosciences); BV650-conjugated anti-CD38 (clone: HB7, Biolegend); BV785-conjugated NKG2D (clone: 1D11, Biolegend). Dead cells were always excluded using a viability dye. Samples were acquired on a LSRFortessa flow cytometer (BD Biosciences) and data were analysed using FlowJo software V10.8.1 (TreeStar). For immunophenotyping, each marker was analysed on the PBMCs of at least 4 different donors.

ILC2 were isolated by Fluorescence Activated Cell Sorting (FACS) on a FACS Aria III (BD) from HC and expanded in StemSpanTM Serum-Free Expansion Medium II (SFEMII, from STEMCELL Technologies) in the presence of IL-2 (100U/ml) and IL-7 (10ng/ml, both from PeproTech).

### EP/ILC2 co-culture assay

ILC2 were stimulated with a cytokine cocktail (IL-2, IP-10, IL-8, IL-6 at 20U/ml, 100ng/ml, 100ng/ml, 20ng/ml, respectively, PeproTech) alone or in combination with EPs isolated from either HC or COVID-19 patients. We set up co-cultures using a concentration of EPs ranging from 2 to 10 μg, that we tested not to kill the cells (data not shown). Supernatants were collected after 48 hours and cytokines were measured using a bead-based immunoassay flow assay, as stated above.

### Statistics

All data are composed of at least three independent experiments. Data were analyzed with Graphpad Prism 9.4.1 for Windows (GraphPad Software, Inc., La Jolla, CA, USA). Due to the small sample size, the data were analyzed using the non-parametric Mann-Whitney test where two groups were compared and the non-parametric Kruskal-Wallis test followed by Dunn’s posthoc test where more than two groups were compared. P-values ≤ 0.05 were considered statistically significant and are indicated in the graphs as reported by the analysis software: *p<0.05, **p<0.01, ***p<0.001, ****p<0.0001.

## Results

### Multiplex protein analysis shows altered EP surface signatures in severe COVID-19 patients and reveals a Co-19-EP identikit

**Figure 1:**
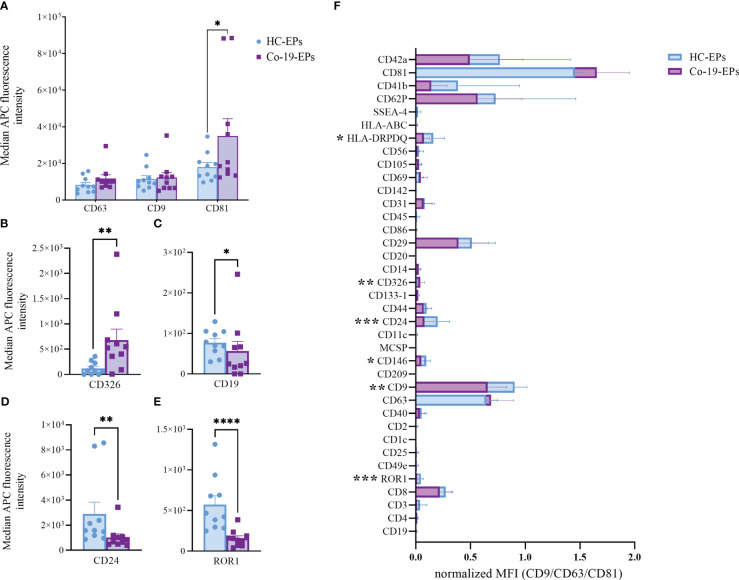
Comparison between EPs from COVID-19 patients (n=10) and HC (n=10) using MACSPlex exosome kit. Background-corrected median APC fluorescence intensity (MFI) for selected markers: **(A)** tetraspanins (CD9, CD63, CD81), **(B–E)** the only significantly different markers between COVID-19 patients and controls (CD326, CD19, CD24, ROR1). **(F)** Superimposed graph with background-corrected median APC fluorescence intensity normalized to exosome marker mean (CD9, CD63, CD81) for all 37 surface epitopes on the different purified EP preparations comparing patients and controls. *p<0.05, **p<0.01, ***p<0.001, ****p<0.001.

To detect any signal produced by cells in the circulation after COVID-19 infection, we firstly evaluated the proteins expressed on the surface of the plasma-derived EPs isolated from COVID-19 patients (Co-19-EPs) and HC (HC-EPs) using bead-based multiplex EV analysis. Considering the overall median fluorescence intensity (MFI) for specific EV markers (i.e., tetraspanins CD63, CD9 and CD81; **Figure 1A**) we observed that only EV-specific tetraspanin CD81 was significantly higher in COVID-19 patients than controls (*P*<0.05). Similarly, the epithelial cell adhesion molecule CD326 (EpCAM) was significantly higher (*P*<0.01) in EPs derived from COVID-19 patients than in controls (**Figure 1B**). Conversely, among differentially expressed epitopes we also found lower expression for CD19, CD24 (B-cell related markers) and ROR1 (stemness marker) in EPs from patients than in controls (**Figures 1C–E**). In particular, most of the immunological-related proteins (CD1c, CD2, CD4, CD11c, CD20, CD25, CD69, CD86, CD209) as well as the hemopoietic marker (CD45) showed either very low expression or were not detected in both groups. Overall, the graph reported in **Supplementary Figure S2** shows the MFI for each marker detected.

Then, we tested the MFI of individual markers after normalization to the mean MFI of the specific EV markers (namely CD9, CD63, and CD81) (nMFI; **Figure 1F**). In addition to the above-described markers (ROR1 and CD24, respectively), we observed that Co-19-EPs differed from controls for the other three markers including CD9 (tetraspanin, *P*<0.01), HLA-DR/DP/DQ (MHC-II, leukocyte, *P*<0.05), and CD146 (endothelial, *P*<0.05). All of them were relatively lower in Co-19-EPs compared to HC-derived EPs. CD326 (EpCAM) expression was detected only on Co-19-EPs (*P*<0.01).

Therefore, these data indicate that a phenotype-based signature on EPs may distinguish severe COVID-19 patients from HC suggesting further investigations on circulating EPs.

### Circulating EPs from severe COVID-19 patients reveal abnormal lipidomic profiling

**Figure 2:**
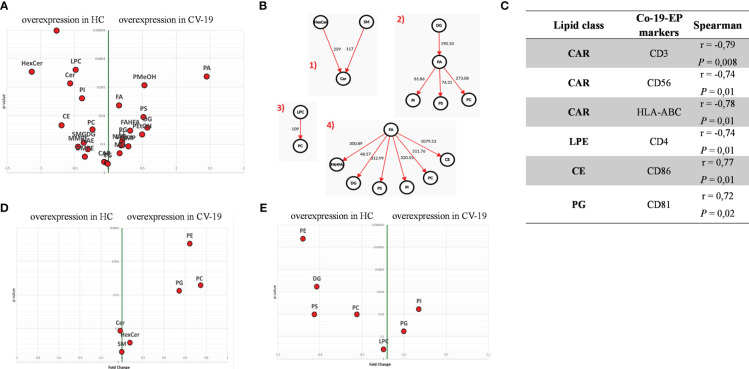
**(A)** Volcano plot of lipid classes. Top left and top right show the lipid classes overexpressed in Co-19-EPs and HC-EPs, respectively. **(B)** Network of transformations between lipid classes: significant changes (*P*<0.05) between lipid classes can be interpreted using known biochemical pathways as reference. Both graphs were made with Excel and Graph Editor from data produced with the lipid class overview fiction of LipidOne. The numbers in the diagram indicate the intensity of the reaction on an arbitrary scale. **(C)** Table reporting the association between lipid classes and MFI of markers on Co-19-EPs using Spearman’s correlation analysis and presented as rank coefficient (r) and P value. Volcano plot of the ratio of oxidized/reduced species **(D)** and the ether/ester-linked ratio **(E)**. The figure shows that the phosphatidylethanolamine (PE), phosphatidylcholine (PC) and Phosphatidylglycerol (PG) classes contain oxidized species and that EPs from COVID-19 patients are enriched in these oxidized species (*P*<0.01). In contrast, EPs from HC are enriched in lipid classes containing ether linkages (PE, diacylglycerol (DG), phosphatidylserine (PS) and PC) (*P*<0.01).

Within EV cargos, lipids are suggested to be involved in EV formation and biological functions (42). To investigate the cargo of circulating Co-19-EPs and eventually how SARS-CoV-2 might influence their cargo, untargeted lipidomic analyses were performed on EPs isolated from the plasma of COVID-19 patients and HC. Lipidomic analysis revealed 1112 lipid species annotated at the molecular species level, grouped into 26 lipid classes. As reported in **Table 2**, we found that almost 70% of the EP-associated lipids are free fatty acids (FA) (28%), cholesteryl ester (CE) (16%), triacylglycerol (TG) (13%) and phosphatidylcholine (PC) (12%). The most significantly different lipid classes between patients and HC were FA, CE, TG, PC, ceramide (Cer), diacylglycerol (DG), lysophophatidylcholine (LPC) and sphingomyelin (SM). Indeed, the volcano plot showed that the amount of SM, HexCer, LPC and Cer is lower (*P*<0.001, respectively) while that of phosphatidylmethanol (PMeHO; *P* = 0.0008) and phosphatidic acid (PA; *P* = 0.0004) is higher in Co-19-EPs compared to HC-EPs (**Figure 2A**). Using known biosynthetic pathways as a reference in **Figure 2B** we show some metabolic pathways activated in COVID-19 patients. Interestingly, we found an inverse correlation between O-acyl-R-carnitine (CAR) and EP markers expressed on Co-19-EPs such as CD3, CD56, and HLA-ABC. Also, lysophosphatidylethanolamine (LPE) reported a negative correlation with the expression of CD4 on Co-19-EPs. By contrast, CE positively correlated with CD86 expression on Co-19-EPs whereas PG lipid class correlated with the exosomal expression of CD81 (**Figure 2C**).

**Table 2:** Summary of lipidomic data.

Class abbreviation	Explained class name	N molecular species	CV19 (nMol/mL)	CV19 SEM (n=10)	HC (nMol/mL)	HC SEM (n=10)	P-value	average Amount (%)
**BMP**	Bismonoacyl glycerophosphate	3	19.5	2.4	15.9	1.8	0.12	0.18
**CAR**	AcylCarnitine	16	11.9	0.7	12.4	2.3	0.41	0.12
**CE**	Cholesteryl ester	9	1246.8	236.9	2012.0	261.1	0.02	16.16
**Cer**	Ceramide	85	576.5	53.6	852.5	50.6	0.0007	7.08
**CL**	Cardiolipin	14	172.8	9.6	150.0	12.3	0.08	1.60
**DG**	Diacylglycerol	74	807.1	113.4	541.3	59.4	0.02	6.68
**DMPE**	Dimethyl-Phosphatidyl ethanolamine	18	9.9	1.9	12.6	4.0	0.27	0.11
**FA**	Free fatty acid	59	2989.9	94.3	2675.6	50.5	0.004	28.09
**FAHFA**	Fatty acid ester of hydroxyl fatty acid	31	67.3	6.4	53.8	2.8	0.03	0.60
**HexCer**	Hexosylceramide	11	14.3	1.5	31.2	3.8	0.0002	0.23
**LPC**	Lysophophatidylcholine	54	223.5	7.3	312.0	19.5	0.0002	2.65
**LPE**	Lysophosphatidyl ethanolamine	5	3.5	0.2	3.1	0.3	0.11	0.03
**MG**	Monoacylglycerol	3	3.8	0.2	3.4	0.5	0.21	0.04
**MMPE**	monomethyl-phosphatidyl ethanolamine	9	7.6	0.6	10.4	2.2	0.12	0.09
**NAE**	N-acyl ethanolamines	41	111.5	4.1	137.5	24.0	0.15	1.23
**NAOrn**	N-acyl ornitines	20	14.1	0.9	12.2	1.2	0.11	0.13
**PA**	Glycerophosphate	4	119.2	17.1	43.5	8.0	0.0004	0.81
**PC**	Phosphatidylcholine	114	1126.7	61.2	1324	77.7	0.03	12.15
**PE**	Phosphatidyl Ethanolamine	75	120.9	9.1	122.5	18.5	0.46	1.21
**PEtOH**	Phosphatidyl ethanol	29	36	4.9	25.5	3.3	0.04	0.30
**PG**	Phosphatidyl glycerol	16	411.6	20.7	354.5	28.7	0.06	3.80
**PI**	Phosphatidylinositol	20	20.0	1.4	26.1	1.4	0.002	0.23
**PMeOH**	Phosphatidyl Methanol	14	16.9	1.4	11.7	0.4	0.0008	0.14
**PS**	Phosphatidylserine	5	4.5	0.5	3.2	0.3	0.01	0.04
**SM**	Sphingomyelin	26	226.9	12.1	385.7	25.0	1.03E-05	3.04
**TG**	Triacylglycerol	357	1335	186.5	1341.8	209.1	0.49	13.27

The synthetic class name, extended name, number of molecular species annotated, concentration and standard error of the mean (SEM) for both the COVID-19 (CV19, n=10) and HC groups (n=10) of EP samples and p-value of difference are reported for each lipid class. The last column shows the percentage of the mean amount for both groups.

In addition, we identified the presence of oxidized molecular species using the LipidOne analyses. We reported the oxidized/unoxidized species ratio or the Ether/Esters linked ratio within each lipid class. The results are represented with the volcano plot showing that EPs from COVID-19 patients are enriched in selected lipid classes that contain oxidized lipid chains including phosphatidylethanolamine (PE; *P*<0.001), PC and phosphatidylglycerol (PG) (*P*<0.01) (**Figure 2D**). Conversely, considering the Ether/Ester ratio within the 27 lipid classes, only the phospholipids were found to contain ether bonds with enrichment on the PE class in HC (*P*<0.001; **Figure 2E**).

**Figure 3:**
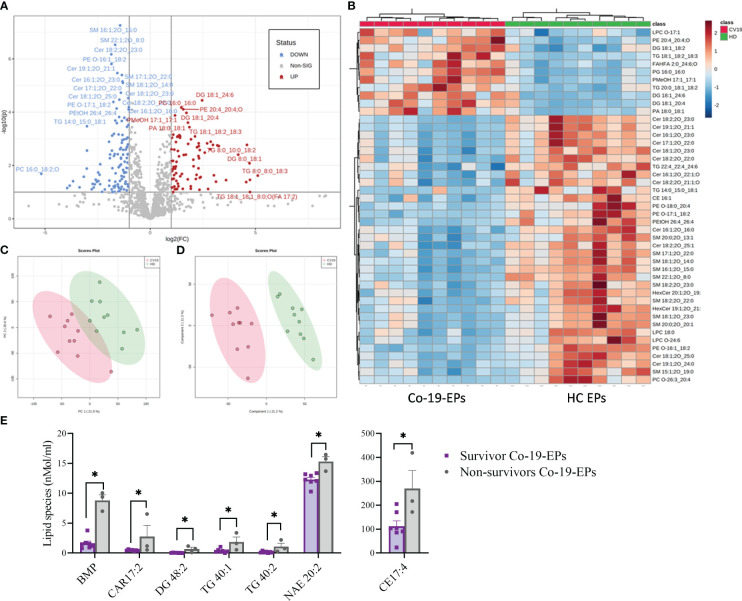
Volcano Plot **(A)**, Heat Map **(B)**, Principal Component Analysis (PCA) **(C)** and Partial least squares-discriminant analysis (PLS-DA) **(D)** of Lipidomic dataset for EPs from COVID-19 patients and HC (n=10, respectively). **(E)** Significant difference ins specific lipid species (namely Bis(monoacylglycero)phosphate (BMP), O-acyl-R-carnitine (CAR)17:2, diacylglycerol (DG) 48:2, triacylglycerol (TG) 40:1, TG 40:2, N-acylethanolamine (NAE) 20:2) between survivors and non-survivors COVID-19 patients (n=7 vs n=3). *P<0.05.

The entire lipidomic dataset was then processed using the MetaboAnalyst platform to perform univariate and multivariate analyses on lipid species. The most prominent observation reported from both the Volcano Plot and the Heat Map (**Figures 3A, B**) was a significant depletion of specific molecular species belonging to the following classes: SM, Cer and some Ether-phospholipids (PE-O) in COVID-19 patients. Indeed, as depicted by the heat map, several sphingolipids (e.g., SM 16:1:20_15:0; SM 22:1:20_8:0, SM 17:1:20_22:0; SM 18:1:20_14:0) were deeply underexpressed in Co-19-EPs. By contrast, several species of diacylglycerols (DG: DG 18:1_24:6/DG 18:1_20:4) and triacylglycerols (TG: TG 18:1_18:2_18:3/TG 20:0_18:1_18:2) were more abundant in Co-19-EPs. Overall data confirmed a pattern that determined the formation of the two clusters as shown in PCA (**Figure 3C**) and PLS-DA (**Figure 3D**) analyses. In both, the first component explains about 21% of the variance in the data. Next, despite the low number of patients, we explored any difference in specific lipids between survivors and non-survivors COVID-19 patients. Of note, we observed specific lipid enrichments discriminating survivors and non-survivors including bis(monoacylglycero)phosphate (BMP) 33:0; O-acyl-R-carnitine (CAR) 17:2; CE 17:4; DG 48:2; N-acylethanolamine (NAE) 20:2 (*P*<0.05, respectively). Also, within TG species, two TGs were significantly up-regulated in non-survivor COVID-19 patients (TG 40:1; TG 40:2, *P*<0.05) (**Figure 3E**).

Overall, we demonstrated that lipidome from EPs distinguishes severe COVID-19 patients from HC.

### Specific lipid species detected in circulating Co-19-EPs correlate with disease aggressiveness scores

To explore the clinical impact of EP lipidome in COVID-19 patients, we investigated whether the lipidome profile of EPs was associated with disease severity parameters including SOFA and PaO_2_/FiO_2_ scores.

As stated above, we observed a strong depletion of SM lipid class in Co-19-EPs (**Figure 3**). Interestingly, we found a negative correlation between SM class and SOFA score (r= -0.82, *P* = 0.009) (data not shown). Taking into account the individual lipid species, Spearman’s correlation analysis revealed that patients with higher SOFA scores had low levels of several lipids’ species including SM 41:1;2O; SM 40:2;3O; SM 42:1;2O; SM 43:1;2O; SM 43:2;2O, monosialodihexosylganglioside (GM3) 38:1;2O and HexCer 42:2;3O. Notably, CL 86:0 reported the strongest negative association with SOFA score (r=-0.93, *P* = 0.0008). By contrast, specific DG and FA were positively correlated with SOFA scores (fatty acid esters of hydroxy fatty acids (FAHFA) 28:4;O/FA 34:0/DG 33:7 DG 36:6) (**Supplementary Table S1**).

Correlation analysis was also performed to detect any association with PaO_2_/FiO_2_ values. Regarding individual lipid species, we observed that FAHFA 22:0;O and two LPCs (LPC O-16:1, LPC O-18:1) were positively associated with PaO_2_/FiO_2_ score. Also, lipid species (SM 41:1;2O; SM 40:2;3O) were both positively correlated to PaO_2_/FiO_2_ score. Of interest, PE 40:3;O and phosphoinositides (PI) O-39:5 levels reported an inverse correlation with PaO_2_/FiO_2_ values (**Supplementary Table S2**).

**Figure 4:**
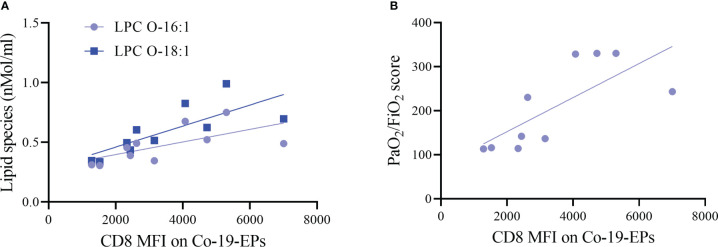
Association between lipid species or clinical score with MFI of markers on Co-19-EPs using Spearman’s correlation analysis. **(A)** Correlations between lipid specie lysophosphatidylcholine (LPC) O-16:1 with CD8 MFI on Co-19-EPs (*P* = 0.01, r = 0.75). A similar association was reported for LPC O-18:1 found in Co-19-EPs and CD8 MFI on Co-19-EPs (*P*=0.0008, r=0.90). **(B)** Correlation between PaO_2_/FiO_2_ score and CD8 MFI on Co-19-EPs (*P*=0.0017, r=0.87).

Next, we investigated any relation between the expression of markers on Co-19-EPs and lipid cargo and clinical features. Both LPC O-16:1 and LPC O-18:1 lipid species, detected in Co-19-EPs, showed a positive association with MFI of CD8 in Co-19-EPs (r = 0.75, *P* = 0.01 and r = 0.90, *P*= 0.0008, respectively). Accordingly, a similar trend was found between CD8 MFI in Co-19-EPs and PaO_2_/FiO_2_ failure score (r = 0.87, *P*= 0.002) (**Figures 4A, B**).

Taken together these data indicate that lipidomic profiling refines the accuracy of disease aggressiveness assessment among severe COVID-19 patients and supports the hypothesis that selective lipid species might act as a prognostic tool for (severe) COVID-19 patients.

### Co-19-EPs reduce the cytokine production ability of ILC2

**Figure 5:**
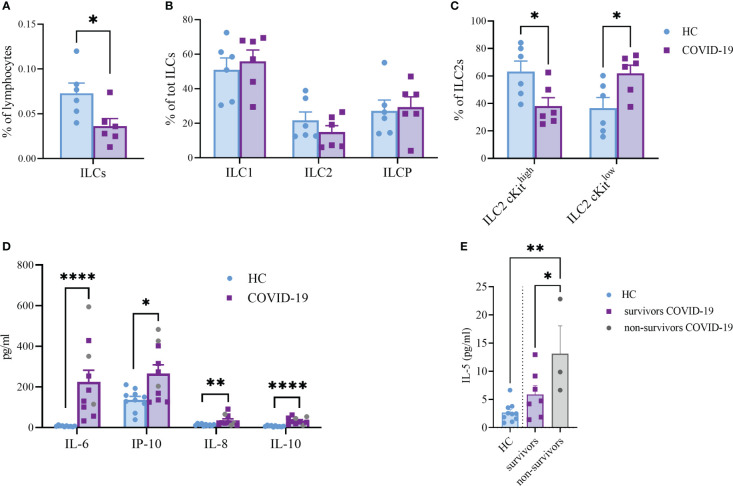
Frequency of total ILC **(A)**, (*P*<0.05), ILC subsets **(B)** and cKit^high^ vs cKit^low^ ILC2 **(C)**, (*P*<0.05) in the circulation of HC and COVID-19 patients (n=6, respectively). Plasma cytokines levels of COVID-19 patients (n=10) and HC (n=10) **(D)**. The plasma levels of IL-6 (*P*<0.0001); IL-10 (*P*<0.0001); IL-8 (*P*<0.01) and IP-10 (*P*<0.05) were significantly increased in COVID-19 patients. Significant difference reported for IL-5 plasma levels between non-survivors (n=3, grey dots) and survivor COVID-19 patients (n=7, *P*<0.05; purple squares) or HC (n=10, *P*<0.01; light blue dots) **(E)**. *p<0.05, **p<0.01, ****p<0.0001

ILC are lymphocytes known to respond to a large variety of stimuli, including cytokines, nutrients, neuropeptides and tumor-derived factors (43, 44). In particular, ILC2 were shown to be sensitive also to lipid mediators and EV stimulation (13, 19). To understand whether ILC2 could respond differently to EPs from COVID-19 patients, we firstly analyzed the profile of circulating ILC of COVID-19 patients and HC. Specifically, as already shown by others, total ILC were decreased in COVID-19 patients in comparison to HC (**Figure 5A**). Although we did not see any significant differences in the ILC subset distribution between COVID-19 patients and HC within the ILC2 subset, we found a significant decrease in the cKit^high^ subpopulation, paralleled with a significant increase in the cKit^low^ population, in COVID-19 patients (**Figures 5B, C**). Because the cKit^low^ population has been proposed to be the ILC2 subpopulation more mature and fully committed (45, 46), our findings suggest that in COVID-19 patients only the ILC2 subset specifically secreting type 2 cytokines is enriched.

**Figure 6:**
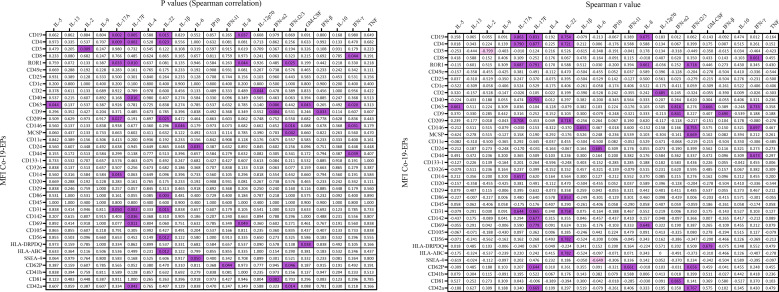
Correlation coefficient matrix heat map reporting any associations of plasma cytokines from COVID-19 patients (n=10). In figure, the color map with double gradient for Spearman correlation coefficient (significant values represented in purple for positive correlations and in light violet for negative correlations).

Next, we evaluated a total of 21 cytokines in plasma samples from patients with severe COVID-19 and HC (**Figure 5D** and **Supplementary Figure S3**). The plasma levels of IL-6 and IL-10 (*P*<0.0001, respectively) were significantly increased in patients with COVID-19 as compared to the HC (**Figure 5D**). Similarly, the levels of IL-8 (*P*=0.005), IP-10 (*P*=0.035) and IL-5 (*P*=0.01; **Figure 5E**) were higher in COVID-19 patients compared to HC. Comparing survivors and non-survivors, only IL-5 plasma levels were significantly increased in non-survivor COVID-19 patients (*P*=0.045) (**Figure 5E**). Furthermore, several Co-19-EP protein markers reported in **Figure 1** were associated with plasma cytokine levels as reported in **Figure 6**. Most of the correlations reported were positive except for those between MFI CD3 with IL-2 and MFI SSEA-4 with IL-6. Of interest, among the significant plasma cytokines detected in COVID-19 patients, IL-8 showed a positive correlation with CD19, CD69 and ROR1 whereas IL-6 positively correlates with MFI CD24. Importantly, MFI CD63 expression on Co-19-EPs is linked to IL-5 plasma levels, the only cytokine different between survivor and non-survivor patients **Figures 5E**, **6**.

**Figure 7:**
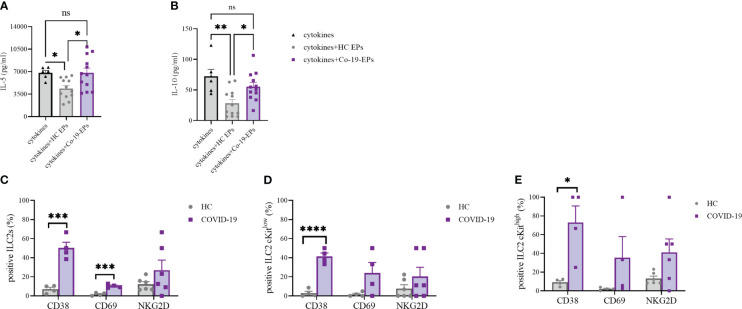
IL-5 **(A)** and IL-10 **(B)** secretion by short-term expanded ILC2 isolated from HC stimulated with cytokines (IL-6, IL-8 and IP-10) alone or in the presence of either HC-EPs or Co-19-EPs. HC-EPs inhibited IL-5 (*P*<0.05) and IL-10 (*P*<0.01) production. **(C–E)** Expression of the activation markers CD38, CD69 and NKG2D in total ILC2 **(C)** and in cKit^low^ **(D)** and cKit^high^ **(E)** ILC2 (4-6 different donors were analyzed for each marker). *p<0.05, **p<0.01, ***p<0.001, ****p<0.0001.

To understand whether the combination of the pro-inflammatory cytokines IL-6, IL-8 and IP-10 together with the EPs isolated from HC and COVID-19 patients could impact the cytokine secretion ability of ILC2, we isolated and expanded human ILC2 from HC *in vitro* and stimulated them with IL-6, IL-8, IP-10 alone or in the presence of either HC-EPs or Co-19-EPs. We found that, while the EPs from HC inhibit IL-5 and IL-10 production, the EPs from COVID-19 patients failed in downregulating these two cytokines, suggesting that the composition of the EPs isolated from COVID-19 patients was supporting the ILC2 activation status (**Figures 7A, B**). Indeed, when we compared the phenotype of ILC2 present in the PBMC of HC and COVID-19 patients, we found that COVID-19 patients’ ILC2 showed a more activated phenotype characterized by an increase in CD38 and CD69 expression and a trend for increased NKG2D (**Figure 7C**). CD38 upregulation was present both in the ILC2 cKit^low^ and cKit^high^ (**Figure 7D–E**).

Altogether these data highlight that the presence of a well-known inflammatory microenvironment in severe COVID-19 patients might be reflected in more activated ILC2 producing high concentrations of both IL-5 and IL-10. Interestingly, at variance with HC-EPs, Co-19-EPs are unable to dampen the activation status of ILC2 in severe COVID-19 patients.

## Discussion

In this study, we identify an EP-associated lipidomic and phenotypic signature of SARS-CoV-2 infected patients with severe disease. Most important, the EP lipid and their protein patterns are associated with the disease aggressiveness scores, highlighting the putative role of Co-19-EPs as a prognostic biomarker cargo in severe COVID-19 patients. Interestingly, critical correlations between EP profile, lipidomic cargo and the immune-inflammatory microenvironment have been found. In addition, these circulating Co-19-EPs are unable to dampen the activated phenotype (as assessed by the ability to produce IL-5 and IL-10) of ILC2 isolated from HC. Despite the limitations of the small number of patients, we developed a valuable method for detecting the effects of SARS-CoV-2 infection and novel insight for studying EPs in infectious diseases.

It has recently been described that EVs might play a role in the host response to SARS-CoV2 infection (47, 48). In the present study, the lipidomic analysis of the EPs from COVID-19 patients enrolled demonstrates the reduced expression of sphingomyelins. Together with glycerophospholipids, sphingolipids are important components of the cell membrane and regulate several processes, such as proliferation and inflammatory responses (49, 50). Moreover, sphingolipid metabolism is involved in exosome secretion (51). Inhibition of SM synthesis has been reported to slow Golgi-to-plasma membrane trafficking of vesicular stomatitis virus G protein, influenza hemagglutinin, and pancreatic adenocarcinoma up-regulated factor suggesting that the SM biosynthetic pathway is broadly required for secretory competence (52). Therefore, SM metabolism can be a potential biomarker for identifying crucial vulnerabilities in COVID-19 patients and a potential target for therapeutic intervention against COVID-19 virus infection. In addition, in this study, Hex Cer and several SM reveal an opposite association with SOFA score which demonstrated that the depletion of sphingolipid species may be closely related to the severity of the disease. We also observe the relative abundance of lipids involved in energy storage, such as triacyl- and diacylglycerols (TG and DG) in EPs from COVID-19 patients. TGs are the most abundant lipids in the human body and are the major source of energy that constitutes a critical component of the lipoproteins (50, 53). Of note, specific TGs (including TG 40:1 and TG 40:2) are selectively increased in non-survivor subjects. In line with this, a myriad of cardiovascular manifestations are observed in COVID-19 patients (54) and, based on the role of lipoproteins in thrombosis, our data may suggest an association between increased TG levels in EPs and cardiovascular events in COVID-19 patients.

The EP surface proteins were also investigated. We found that the exosome markers CD9, CD63 and CD81 are present in EPs isolated from all groups. Among differentially expressed proteins, CD24, CD146 and CD326 show remarkably higher expressions in EPs from COVID-19 patients.

CD24 is highly expressed by immune cells and cancer cells and it is known to play an inhibitory role in B-cell activation responses and the control of autoimmunity (55). It has recently been described that CD24 stimulation of B cells may trigger a transfer of receptors functional in recipient cells *via* EVs (56).

CD146, a membrane and immunoglobulin superfamily protein that is normally expressed by endothelial cells and Th17 cells, promotes the adhesion, rolling and extravasation of lymphocytes and monocytes across the endothelium. Indeed, functionally, CD146 is involved in angiogenesis and inflammation (57, 58).

Finally, CD326 is an adhesion molecule that is characteristic of some epithelia and many carcinomas and has been implicated in intercellular adhesion and metastasis (59). It has recently been described to play a role in coagulopathy (60, 61). Overall, the phenotype of circulating EPs from severe COVID-19 patients suggests that the hyperexpression of these EV biomarkers might contribute to affecting the immune response and the inflammatory microenvironment. For instance, it is worth noticing that it has been previously reported a link between the LPC and T cell homeostatic turnover (62). Herein, we find a direct association between Co-19-EPs expressing CD8 and two specific lipid species (LPC O-16:1 and LPC O-18:1), suggesting a defective role in the release of EV by CD8^+^ memory T cells in COVID-19 patients (62). This hypothesis is also confirmed by a corresponding association with the PaO_2_/FiO_2_ failure score that show a more aggressive disease in the patients with lower MFI for CD8 in Co-19-EPs.

The challenge of the COVID-19 pandemic is to predict intensive care admission or death of COVID-19 patients. Based on the explorative data of this study, in-depth phenotype and lipidome profiling of EP could be considered novel tools for better stratification of the patients, helping selection and decision for clinical studies, or avoiding the risk of therapy-related complications. Since few data concerning the role of EPs and their lipid-associated cargo in COVID-19 are available, further studies, combining lipidomic data with biological and immunological characterization, may help to elucidate specific (immuno) pathogenetic mechanisms and identify novel treatment strategies for virus infections. Importantly, considering EV trafficking, the lipid composition of EV membranes may play a role in the stability of these vesicles as well as facilitating binding to and uptake into recipient cells such as immune cells.

Along with lipidomic and surface protein expression, we also performed co-culture experiments with circulating EPs from HC or COVID-19 patients using ILC2 as a target. In line with others, we find that the ILC2 from severe COVID-19 patients show a more activated phenotype in terms of CD38, CD69 and a trend for NKG2D expression; the latter marker was already shown to be upregulated in patients showing no need for mechanical ventilation and a shorter hospitalization (63). Our data suggest that the activated phenotype of ILC2 as well as the higher capacity of producing IL-5 and IL-10 might be linked with the different cargo of the circulating EPs. Indeed, only HC-EPs are efficient in suppressing the ILC2 cytokine secretion capacity, while Co-19-EPs lose this property. Whether this inhibitory capacity is due to the different lipidic or protein composition of the EPs is yet to be investigated. Overall, although EVs may represent a mechanism by which the SARS-CoV-2 escapes the immune system, our data indicate that circulating EPs may alarm the innate immune system by modifying the production of inflammatory cytokines. These findings shed light on the diverse effects of circulating EPs on the inflammatory/immune response of COVID-19. Consistently, we found the plasma levels of IL-5, IL-6, IL-8, IL-10 and IP-10 to be significantly higher in severe COVID-19 patients compared with control plasma. Previous data identified IL-10 and IP-10 as putative biomarkers associated with poor outcomes. In this regard, IL-10 has been shown as a putative regulator of COVID-19 pathogenesis in association with IL-6 (64), whereas IP-10 has been investigated for its role in thrombosis in COVID-19 patients (65). For instance, IP-10 is secreted by many cell types in response to interferon-gamma IFN-including monocytes, endothelial cells and fibroblasts (66) and acts as a chemotactic agent for immune cells such as T cells, NK cells, monocytes/macrophages and dendritic cells (65). In addition, IL-6, TNF-α and IL-8 were considered strong and independent markers for patient survival (67–69). Notably, Li L et al. showed both IL-8 and IL-6 as biomarkers of disease prognosis for COVID-19 patients suggesting them as putative therapeutic targets (69). Of interest, IL-5 plays a crucial role in our cohort being significantly different not only between HC and COVID-19 patients but also between non-survivor and survivor patients. It is already known the role of IL-5 in the growth, survival, and activation of eosinophils (70). Despite we do not find any association between IL-5 and the absolute eosinophil count of our patients (data not shown), our results suggest that the Type 2 immune response is involved and may be aggravated by SARS-CoV-2-induced pneumonia (70). Following previous work (71), EVs from subcutaneous immunotherapy-treated mice exert effects on IL-5 production from ILC2 suggesting novel therapeutic options using EVs. Indeed, it has been also demonstrated the potential for using EVs as powerful and feasible cargo for drug delivery (72). The natural origin of EVs enables them to reduce immunogenicity compared with existing delivery systems. Thus, an EV-based drug delivery system may be an attractive candidate to manipulate also the cytokines secretion by specific cell subsets for a novel effective treatment for COVID-19. Overall, our data on circulating cytokines confirm and highlight the complex immune/inflammatory network of COVID-19 pathogenesis and suggest that blocking one cytokine alone could be an ineffective strategy (64, 67, 73).

At last, even though these findings depict an “EP signature” of severe COVID-19 patients, it should be highlighted that at the time of sample collection severe COVID-19 patients were under treatment; therefore, we can not rule out the possibility that treatment might have influenced the EP pattern.

In summary, this study demonstrates that a distinct lipidomic and phenotypic signature characterizes EPs in severe COVID-19 patients. In addition, this study shed light on the mechanisms by which circulating EPs modulate the innate immune response. With the limitations related to the small cohort of COVID-19 patients included, these findings might have the potential for prognostic implications of EPs in severe COVID-19 patients. Since future and innovative therapeutic approaches in the COVID-19 current scenario may rely on signals carried by Co-19-EPs, our data represent a step toward the identification of a Co-19-EP-specific pattern of secret signals released in circulation in COVID-19 patients.

## Data availability statement

The raw data supporting the conclusions of this article will be made available by the authors, without undue reservation.

## Ethics statement

The studies involving human participants were reviewed and approved by Comitato Etico di Area Vasta Emilia Centro della Regione Emilia-Romagna (CE-AVEC) (377/2020/Oss/AOUBo). The patients/participants provided their written informed consent to participate in this study.

## Author contributions

DF, ST and LC contributed to the conception, research design and drafting of the paper and conceptualization methodology. TT and GC collected human samples. RP, HA, SB and LU performed the LC-MS/MS measurements and analysis. DF, RP, ST, MCe, GN and HA performed data curation. TT, GC, GM and VR provided clinical data and enrolled patients for the study. DF, FR PT performed the flow-cytometry analysis. DF, ST, MCe, CM, CE, CJ, MC, LC and FP reviewed and edited the manuscript. DF, MCa, LC, and FP provided supervision of the study. All authors contributed to the article and approved the submitted version.
