# Supplementary material for: Circulating extracellular particles from severe COVID-19 patients show altered profiling and innate lymphoid cell-modulating ability
Source: Front Immunol. 2023 May 3;14:1085610. doi: 10.3389/fimmu.2023.1085610 (PMC10189636; doi:10.3389/fimmu.2023.1085610)
Supplement: Supplementary file 1 [file DataSheet_1.docx]

**SUPPLEMENTARY MATERIALS**

**Supplementary Figure S1:**

**
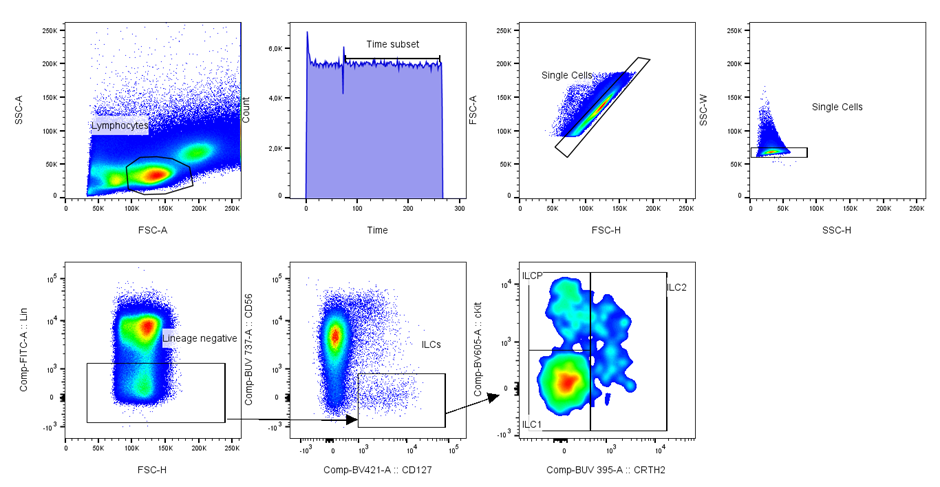
**

ILC identification by FACS. ILCs are gated in the lymphocyte region, after the removal of time effects and doublets, as living Lineage (CD3, CD4, CD8, CD14, CD15, CD16, CD19, CD20, CD33, CD34, FceRI, CD203c) negative, CD56 negative, CD127 positive cells. From total ILCs, ILC1s are identified as CRTH2-cKit-, ILCPs as CRTH2-cKit^+^, ILC2s as CRTH2^+^cKit^+/-^.

**Supplementary Figure S2:**


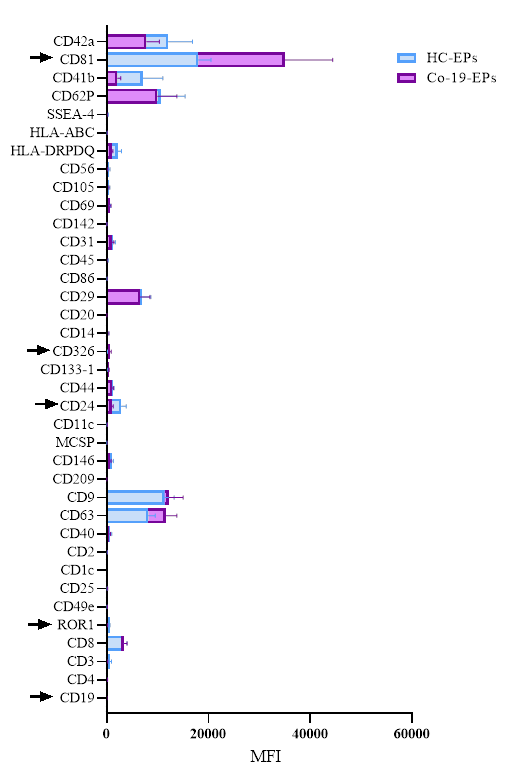


Comparison between EPs from COVID-19 patients (n=10) and HC (n=10) using MACSPlex exosome kit. Background-corrected median APC fluorescence intensity (MFI) for 37 markers in a superimposed graph. Significant markers are reported with an arrow and shown in Figure 1A-D.

**Supplementary Figure S3:**

Plasma cytokines’ levels of COVID-19 patients (n=10) and HC (n=10). The plasma levels of IL-1β, TNF-α, IFN-λ1, IFN-β, IL-13, IL-12p70, IFN-α2, IL-4, IFN-λ2/3, IL-9, GM-CSF, IL-17A, IL-17F, IL-22 and IL-2 were not different between the 2 groups.

**Supplementary Table S1:**

| **Lipid species** |  | **SOFA score** |
| --- | --- | --- |
| **CL 86:0** | r  *P* | -0.93  0,0008 |
| **DG 33:7** | r  *P* | 0.92  0.0008 |
| **DG 36:6** | r  *P* | 0.85  0.005 |
| **FA 34:0** | r  *P* | 0.82  0.009 |
| **FAHFA 28:4;O** | r  *P* | 0.96  0.0004 |
| **GM3 38:1;2O** | r  *P* | -0.85  0.005 |
| **HexCer 42:2;3O** | r  *P* | -0.82  0.007 |
| **FA 18:0;2O** | r  *P* | -0.83  0.007 |
| **SM 43:1;2O** | r  *P* | -0.85  0.005 |
| **SM 43:2;2O** | r  *P* | -0.86  0.004 |
| **SM 41:1;2O** | r  *P* | -0.87  0.003 |
| **SM 40:2;3O** | r  *P* | -0.85  0.005 |
| **SM 42:1;2O** | r  *P* | -0.85  0.005 |
| **SL 47:3;0O** | r  *P* | -0.82  0.009 |

Table for the association between lipid species with SOFA score using Spearman's correlation analysis.

**Supplementary Table S2:**

| **Lipid species** |  | **PaO_2_/FiO_2_ score** |
| --- | --- | --- |
| **FAHFA 22:0;O** | r  *P* | 0.85  0,002 |
| **LPC O-16:1** | r  *P* | 0.86  0.001 |
| **LPC O-18:1** | r  *P* | 0.86  0.002 |
| **PE 40:3;O** | r  *P* | -0.81  0.006 |
| **PI O-39:5** | r  *P* | -0.79  0.008 |
| **SM 40:2;3O** | r  *P* | 0.74  0.01 |
| **SM 42:1;2O** | r  *P* | 0.74  0.01 |

Table for the association between lipid species with PaO_2_/FiO_2_ score using Spearman's correlation analysis.
